# GFRA1: A Novel Molecular Target for the Prevention of Osteosarcoma Chemoresistance

**DOI:** 10.3390/ijms19041078

**Published:** 2018-04-04

**Authors:** Mihwa Kim, Dae Joon Kim

**Affiliations:** Department of Biomedical Sciences, School of Medicine, University of Texas Rio Grande Valley, Edinburg, TX 78541, USA; mihwa.kim@utrgv.edu

**Keywords:** GFRA1, osteosarcoma, chemoresistance, GDNF, RET

## Abstract

The glycosylphosphatidylinositol-linked GDNF (glial cell derived neurotrophic factor) receptor alpha (GFRA), a coreceptor that recognizes the GDNF family of ligands, has a crucial role in the development and maintenance of the nervous system. Of the four identified GFRA isoforms, GFRA1 specifically recognizes GDNF and is involved in the regulation of proliferation, differentiation, and migration of neuronal cells. GFRA1 has also been implicated in cancer cell progression and metastasis. Recent findings show that GFRA1 can contribute to the development of chemoresistance in osteosarcoma. GFRA1 expression was induced following treatment of osteosarcoma cells with the popular anticancer drug, cisplatin and induction of GFRA1 expression significantly suppressed apoptosis mediated by cisplatin in osteosarcoma cells. GFRA1 expression promotes autophagy by activating the SRC-AMPK signaling axis following cisplatin treatment, resulting in enhanced osteosarcoma cell survival. GFRA1-induced autophagy promoted tumor growth in mouse xenograft models, suggesting a novel function of GFRA1 in osteosarcoma chemoresistance.

## 1. Introduction

**Figure 1:**
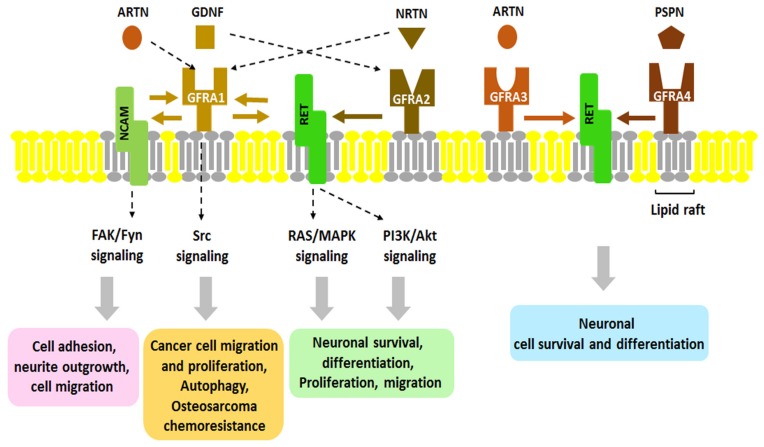
The interaction of glial cell line-derived neurotrophic factor (GDNF) family ligands (GFLs) with their receptors. Four homodimeric GFLs (GDNF, NRTN, ARTN, and PSPN) recognize their corresponding GDNF family receptor α (GFRα/GFRA1-4). GDNF specifically binds to GFRA1, NRTN to GFRA2, ARTN to GFRA3, and PSPN to GFRA4. Dotted arrow indicates cross-interaction of GFLs with GFRA which has been observed. Binding of GFL with GFRA activates protein tyrosine kinase RET. Neural cell adhesion molecule (NCAM) has been identified as an alternative signaling receptor for GFLs. RET also has been known as the representative receptor tyrosine kinases (RTKs) for these neurotrophic factor receptors. Activation of RET or NCAM via the GFL/GFRA complex is involved in various physiological functions, such as neuronal cell growth, differentiation, migration, and osteosarcoma chemoresistance via autophagy. Gray-colored area of the membrane indicates lipid raft. NRTN, neurturin; ARTN, artemin; PSPN, persephin.

**Figure 2:**
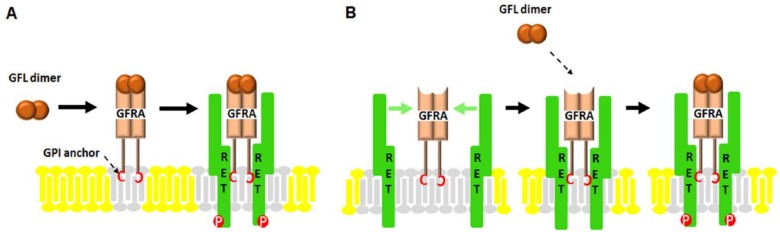
Two hypothetical modes of RET interaction/stimulation by GFL/GFRA. The extracellular domain of RET tyrosine kinase binds with GFL/GFRA which is linked to the plasma membrane via a glycosyl phosphatidylinositol (GPI) anchor. The interaction of RET with GFL/GFRA leads to the phosphorylation of tyrosine residues located in intracellular domain of RET and activates downstream signaling pathways. (**A**) GFL dimer binds to the GPI-anchored GFRA in lipid rafts and recruits RET; (**B**) GFRA without ligand forms a complex with RET that can serve as a receptor for GFL dimer.

**Figure 3:**
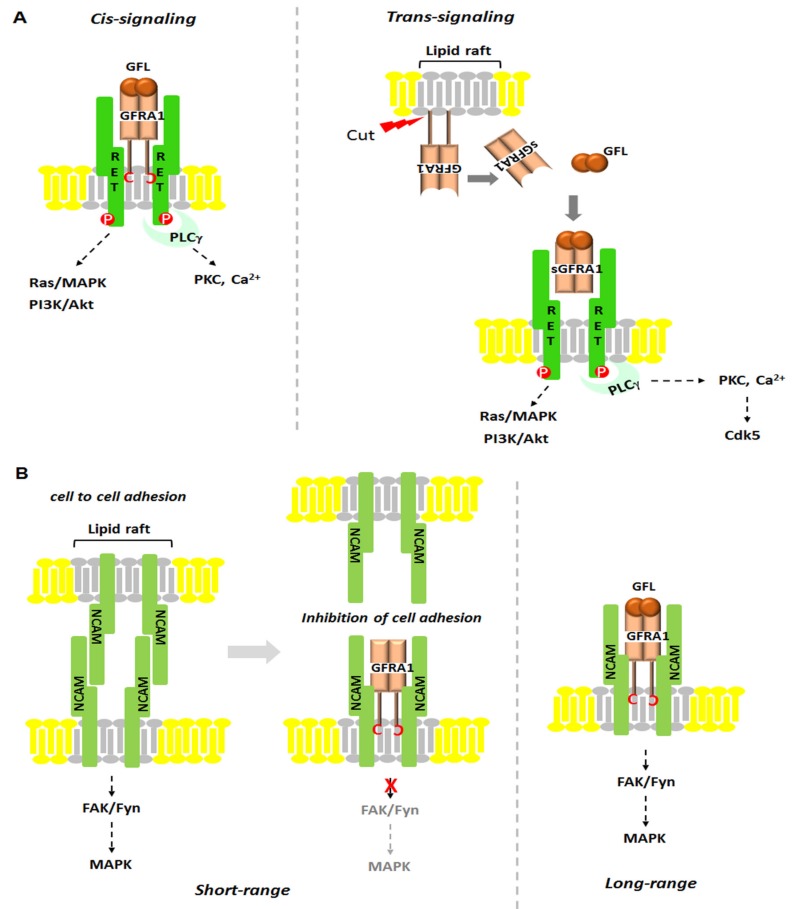

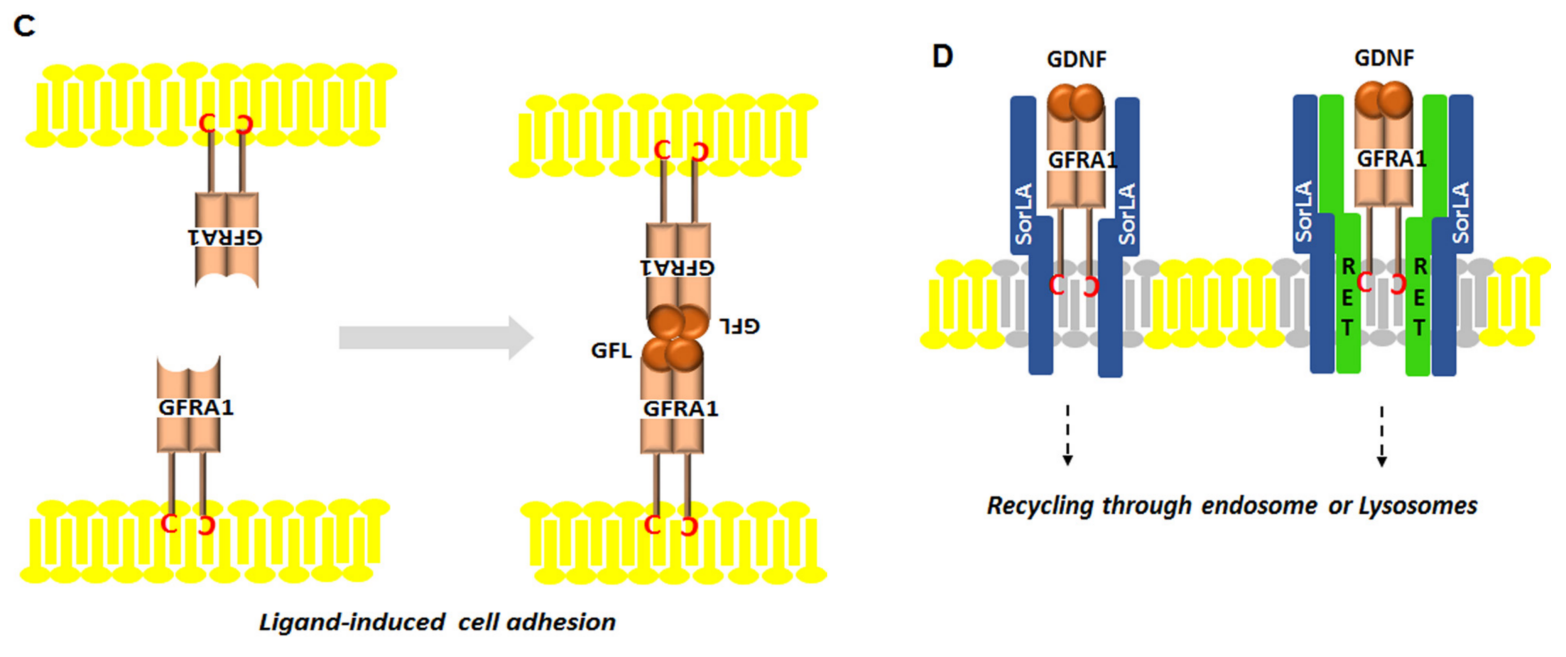
Schematic presentation of regulatory mechanisms of GFL-GFRA1 signaling. (**A**) RET is recruited to the lipid microdomain (or lipid rafts) by GPI-anchored GFRA1 (left side, cis-signaling) and by soluble GFRA1 (right side, sGFRA1, trans-signaling) released from plasma membrane. During cis-signaling, GFRA1 is anchored to lipid rafts by GPI, allowing GFLs to bind to GFRA1 to transduce their signals to co-receptor RET which can then activate downstream targets like Src kinase, PLCγ, or PI3K/AKT. Trans-signaling occurs when the GDNF/sGFRA1 complex binds to RET on another peripheral neuron which does not express GFRA1. GDNF/sGFRA1/RET can then transduce their signal to downstream targets such as PI3K/AKT and PLCγ/Cdk5, resulting in neuronal survival and neurite outgrowth; (**B**) In the absence of GFLs, short-range signaling by homophilic interactions between p140^NCAM^ can be disrupted by the presence of GFRA, resulting in the inhibition of cell adhesion, or extracellular signaling crosstalk (left side). In the presence GFLs, the association of membrane-bound GFRA with p140^NCAM^ results in the activation of the Fyn-FAK-MAPK signaling pathway (right side); (**C**) Ligand-induced cell adhesion; (**D**) Recycling of GDNF/GFRA1/RET complex by SorLA. SorLA/GFRA1-mediated GDNF uptake occurs by RET-independent endocytosis. SorLA/GFRA1 complex directs GDNF to lysosome. SorLA/GFRA1-mediated RET endocytosis is independent of GDNF.

Neurotrophic factors (NTFs) are a family of peptides or small proteins including neurotrophins, neurokines and the glial cell line-derived neurotrophic factor (GDNF) family of ligands, or GFLs. NTFs are known as key regulators of neuronal ontogeny, neuronal survival, differentiation, migration, synapse plasticity and function in both the developing and adult nervous systems. They exert their signaling through kinases such as receptor tyrosine kinases (RTKs). Since it was characterized in 1993, the GDNF subfamily of NTFs has been well-studied. The GDNF family consists of GDNF, Neurturin (NRTN), Artemin (ARTN), and Persephin (PSPN) which are also members of the transforming growth factor-beta superfamily [1,2,3,4,5,6,7]. GDNF was isolated from a glial cell line related to the survival of midbrain dopaminergic neurons, and its loss is associated with neurodegenerative diseases like Parkinson’s disease [8]. Mainly, GDNF affects many types of neurons in both the peripheral (sympathetic, parasympathetic, sensory and enteric neurons) and central nervous systems (midbrain dopamine neurons and motoneurons) [9,10]. RET (rearranged during transformation) receptor tyrosine kinase was the first RTK to be identified as a GFL co-receptor in 1996. RET is a single transmembrane-spanning RTK that is essential to vertebrate development of the central and peripheral nervous systems, kidney and Peyer’s patch organogenesis, and spermatogenesis [11]. Its mutated form is found in endocrine human cancers and cases of Hirschsprung disease [12]. However, it was thought that RET cannot bind directly to GDNF until another GFL co-receptor, GDNF receptor alpha 1 (GFRA1), was identified. Interestingly, the ligand-binding specificity of GFLs is determined by GFRA proteins that have unique high binding affinities for each GFL. So far, three glycosylphosphatidyl inositol (GPI)-anchored co-receptors related to GFRA1 have been found: GFRA2, GFRA3 and GFRA4 (Figure 1) [9]. As shown in Figure 1, GFRA1 directly interacts with GDNF and alternatively, it can bind ARTN or NRTN; GFRA2 binds with NRTN; GFRA3 interacts with ARTN; and GFRA4 is the preferential co-receptor for PSPN. Although it has been shown that GFLs bind with their co-receptors, the mechanisms by which these interactions occur still have not been elucidated. As described in Figure 2, there are currently two hypothetic modes of interaction between GDNF and RET. In the first mode, GFLs make dimers and form a high-affinity complex with one of four GFRAs. Each GFL-GFRA homodimer complex recruits c-RET to a lipid raft, a platform containing receptors and messenger proteins for signal transduction, which triggers autophosphorylation of specific tyrosine residues in the kinase domains of RET and intracellular signaling [9]. In the second mode, a pre-associated complex between GFRA and RET could form the binding site for the GFLs [13]. The physiological roles and regulatory mechanism of GDNF has been relatively well studied as opposed to ARTN, NRTN and PSPN which have rarely been reported. As shown in Figure 3, once GDNF/GFRA1/RET makes a complex, it activates various signaling pathways, such as RAS/MAPK, PI3K/Akt and PLCγ pathway, which contribute to cell proliferation, adhesion, migration, differentiation and survival and which contribute to the pathogenesis of both cancer and neuronal disease [14,15]. However, in a RET-independent pathway, GPI-anchored GFRA1 is localized to lipid rafts in the plasma membrane and then, the GDNF-GFRA1 complex triggers a Src-dependent signaling pathway, leading to neuronal differentiation and survival [13]. Once RET was identified, additional attempts to find other signal-transducing GDNF receptors failed for several years until the Paratcha research group discovered neural cell adhesion molecule (NCAM) as an alternative signaling co-receptor for GFLs [16]. Indeed, in cultured Schwann cells and cortical neurons, GDNF/GFRA1/p140^NCAM^ complex activates the cytoplasmic protein tyrosine kinase Fyn and FAK, two downstream mediators of p140^NCAM^ [16].

Until now, GDNF/GFRA1 signaling has been regarded as an emerging potential therapeutic target in neuronal disease. However, recent studies have reported that GFRA1 also is implicated in cancer signaling. Moreover, our recent research with osteosarcoma demonstrated that GFRA1 promotes cell survival against the well-known anticancer agent cisplatin by promoting autophagy, a novel resistance mechanism against chemotherapy. In this review, we summarize the current studies and focus on the physiological role of GFRA1 in chemoresistance.

## 2. Regulatory Mechanisms of GDNF/GFRA1 Signaling

Since GDNF was initially identified as a survival factor for midbrain dopaminergic neurons, it has been investigated in regards to various neural developmental processes, including kidney morphogenesis and spermatogenesis [11,17,18,19]. GDNF signaling is much more complex than initially expected due to the complexity of interaction among GDNF and its co-receptors. This section will briefly summarize the regulatory mechanisms for GDNF signaling and its receptors.

### 2.1. GFRA1-RET Signaling

GFRA1-RET signal transduction can be divided into two pathways: cis-signaling and trans-signaling. As described in Figure 3A, *cis*-signaling is an action mechanism that occurs on the same membrane. GPI-anchored GFRA1 is localized to a specialized plasma membrane microdomain, or lipid rafts. Once GFLs bind to GFRA1, it transfers its signals to co-receptor RET which can activate downstream targets like Src kinase, RAS/MAPK, PLCγ, or PI3K/AKT, resulting in cell proliferation, migration and survival in cancer and neuronal cells [20]. Trans-signaling is referred to as non-cell-autonomous action at a distance (Figure 3A). Soluble GFRA1 (sGFRA1) is constitutively released by phospholipases and shed from the cell surface. sGFRA1 binds to GDNF with high affinity and is stably-floated. Once in complex, sGFRA1/GDNF promotes neuronal survival and neurite outgrowth by binding with RET on the membrane of GFRA1-deficient RET-expressing cells [21]. The discovery of sGFRA1 in both Schwann cells and injured sciatic nerves opens the possibility that lesioned peripheral nerves can be readily recovered through GDNF/sGFRA1/Cdk5 axis-axon-guidance activities provided by the adjacent Schwann cells [21,22].

### 2.2. GFRA1-NCAM Signaling

Neural cell adhesion molecule (NCAM) is a hemophilic binding glycoprotein expressed on the surface of neurons, glia and skeletal muscle [23,24,25,26,27,28]. It also has been found to be expressed in the immune cells including natural killer cells, gamma delta T cells, activated CD8^+^ T cells and dendritic cells [29,30,31]. Alternative mRNA splicing of the NCAM gene produces three isoforms: p120, p140, p180 [32]. Studies have shown that NCAM plays critical roles in cell-cell adhesion, neurite outgrowth, neuronal survival, synapse formation and synaptic plasticity [16,33,34]. Interestingly, the presence of GDNF/GFRA1 is required for NCAM-mediated neuronal signal transduction. Given that NCAM is an alternative signaling receptor for GFLs, recent investigations of NCAM have been focused on the clarification of novel mechanisms that could be applied towards successful clinical trials using GDNF or GFRA1 [16]. Homophilic binding occurs between NCAM molecules on opposing surface (*trans*-) and on same surface (*cis-*). As shown in Figure 3B, dimerization of NCAM functions in neurite growth via FAK and Fyn, two mediators downstream of NCAM [16]. As a negative regulator, GFRA1 can inhibit the interaction between NCAM molecules in the absence of GDNF (Short-range). However, once GDNF is infused from outside, GFRA1 promotes extracellular crosstalk by helping GDNF to bind with NCAM (Long-range). As will be described in the next regulatory mechanism, in vivo the NCAM-deficient model shows abnormality of size in the olfactory bulb (OB) caused by defects in neuronal cell migration in the rostral migratory stream (RMS). Interestingly, overexpression of GDNF or NCAM in RET-deficient mice recover the size of OB. The results indicate that RET-independent GDNF signaling via NCAM can play a role in the migration and guidance of neuroblasts and in the development of the RMS-OB [16].

### 2.3. GDNF-GFRA1 Signaling: Ligand-Induced Cell Adhesion

Unlike other cell-adhesion molecules (CAMs) trans-interacting among cells, GFRA1-mediated cell adhesion requires the presence of GDNF. As such, GFRA1 is sometimes referred to as a ligand-induced cell-adhesion molecule (LICAM). In this case, GPI-anchored GFRA1 and soluble GDNF form a complex that induces cell adhesion working in a similar status (Figure 3C). Although the means by which GDNF-induced trans-homophilic interactions occur are not clear, GFRA1-knockout mutants indicate that GDNF-induced cell adhesion is required for the linkage between GFRA1 and GDNF. Thus, this mechanism plays a role in the development of the nervous system by permitting the interaction between neuronal and glial cells [35].

### 2.4. GFRA1-SorLA Signaling 

Until now, the mechanism of GDNF downregulation has not been studied, unlike degradation by ubiquitination. Recently, the Glerup group demonstrated that SorLA (sorting protein-related receptor) binds to GFRA1 or RET, which induces GDNF/GFRA1 recycling (Figure 3D). In this process, SorLA plays a role as a co-receptor of GDNF. Indeed, SorLA has much higher affinity for GDNF/GFRA1 than RET for endocytosis, but it does not degrade GFRA1. While SorLA mediates GFRA1 recycling through the endosome, GDNF is transferred to the lysosome for degradation. In SorLA-deficient mice, the level of GDNF protein is elevated with abnormal GDNF activity [36]. The findings showed a novel role for GFRA1 in that it is involved in the degradation of GDNF through its binding to SorLA, which induces GDNF/GFRA1 recycling through endocytosis.

## 3. Physiological Roles of GFRA1 in Disease

GDNF was purified and characterized as a survival factor of the embryonic dopaminergic neurons of the midbrain, which degenerate in Parkinson’s disease [8]. In addition, it works as a potent neurotrophic factor for spinal motor neurons [37]. Thanks to its robust role in the neuron, GDNF is regarded as a highly promising therapeutic agent for the treatment of neurodegenerative disease. Although phase I clinical trials with Parkinson’s patients using direct infusions into the putamen were very effective, phase II studies resulted in conflicting outcomes [38,39]. As previously mentioned, GFRA1 is a GPI-anchored cell surface receptor for GDNF, NRTN and ARTN. The effect of GFRA1 signaling can be determined by a variety of interactions and its expression levels. In other words, its aberrant expression or mutation of co-receptors may cause the abnormalities in neuronal survival and differentiation. Thus, the key to combating GFL/GFRA1-related disease is to understand the widespread expression of GFRA1 proteins in various tissues in combination with and without RET signaling. 

In certain types of neurons, the RET-associated GFL/GFRA1 complex activates several intracellular signaling cascades, which regulate cell survival, differentiation, proliferation, migration, chemotaxis, branching morphogenesis, neurite outgrowth and synaptic plasticity. Our previous report demonstrated that GFRA1 overexpression is associated with advanced pancreatic cancer [40]. APE1 (apurinic/apyrimidinic endodeoxynuclease 1)-induced GFRA1 protein forms a complex with RET through a lipid raft, which stimulates cell proliferation and migration in pancreatic cancer. Additionally, GFRA1 expression increases neurite outgrowth and survival against β-amyloid peptide accumulation in neuronal cell lines [41]. In other words, loss of GFRA1 is implicated in neurodegenerative disease, such as Alzheimer’s (AD) and Parkinson’s disease (PD) [41]. Mutations in RET are common in Hirschsprung’s disease which is characterized by the absence of neuronal ganglia in various parts of the colon, leading to severe constipation and intestinal obstruction during childhood [42]. Although a GFRA1 mutation has not been found in Hirschsprung’s patients, Hirschsprung’s disease has been observed in heterozygous *GDNF+/−* mice [43]. However, mutated GDNF does not reduce the activation of RET [44]. Interestingly, mutated NRTN has also been shown to be involved in this disease. Collectively, the results imply that mutation in GDNF, NRTN or RET reduce the affinity for GFRA1 interaction, resulting in Hirschsprung’s disease [43,45,46].

In the RET-independent pathway, there are two possibilities for GFRA signaling: (1) use of other receptor systems (NCAM, SorLA, Syndecan, MET, ErbB4) or (2) direct GFL/GFRA1 signaling through cytosolic tyrosine kinases, like Src [47,48,49]. For example, GDNF/GFRA1 signaling via NCAM contributes to the regulation/modulation of the process of neuronal development, including proliferation, neuronal fate specification, differentiation, neurite outgrowth, and synapse formation [50,51,52,53]. In contrast, GFRA1 can act to antagonize NCAM-mediated cell adhesion, which directly acts between GFRA1 and the 4th domain of NCAM [54]. Independently of NCAM, GFRA1 can also act as a cell adhesion molecule by itself, which is termed ligand-mediated cell adhesion [35]. Syndecan-3 is implicated in GDNF signaling through its heparan sulfate chains [47]. In GABAergic interneurons of the medial ganglionic eminence (MGE), GDNF signaling can transfer via MET or ErbB4 [48,49]. 

As mentioned above, most studies of GDNF/GFRA1 signaling are related to neurodegenerative (Parkinson’s disease and Alzheimer’s disease) and neuropsychiatric diseases (addiction, bipolar disorder, obsessive compulsive disorder, depression, anxiety, autism, schizophrenia, and attention deficit hyperactivity disorder). Moreover, recent therapeutic approaches for GDNF/GFRA1 signaling are intended to investigate how GDNF is infused to target cells or to find what can interact with GFRA1 [55]. In the brain dopamine system, GDNF-based therapy is rising in treatment of Parkinson’s disease (PD) which is caused by cell death in the substania nigra pars compata (SNpc) of the midbrain, due to loss of dopamine [56]. SNpc contains about 10,000–15,000 dopaminergic neurons which express GFRA1 and RET [57]. Recent PD models show the symptom after more than 50% of striatal dopamine levels and 30–40% of SNpc neurons are lost [58]. Additionally, rodent models of PD demonstrate that the axonal trees of the remaining axons can re-innervate the striatal areas left vacant by the degenerating dopaminergic neurons to inhibit or reverse the progression of PD via GDNF delivery [58]. Moreover, the recent rodent models for ectopic overexpression of GDNF or GFRA1 in the forebrain of mice using a neuron-specific CAMKII promoter demonstrates that endogenous GDNF is essentially required for midbrain dopaminergic neuron development of maintenance [59,60]. Subsequently, the successes and draw-backs of GDNF-based therapies in PD need a physiological understanding of GDNF/GFRA1 signaling in vivo. 

GDNF and its receptors have been shown to be concentrated within the midbrain. However, they are often observed in many other brain regions in the CNS. Recent studies show that GDNF and its two receptors (GFRA1 and NCAM) are expressed by hippocampal neurons during embryonic and early postnatal stages [22,35,61]. In other words, GFRA1 functions as a ligand-induced cell adhesion molecule at synaptic sites, which promotes cell-to-cell interaction for communication among cells. Once abnormal expression of GFRA1 or NCACM decreases presynaptic maturation during hippocampal synaptogenesis, neuropsychiatric disorders can occur [22,61]. In GABAergic neurons, GFRA1 signaling plays a critical role in the development of the olfactory system. Although GFRA1 itself was not expressed by matured GABAergic neurons in the olfactory bulb (OB), all major classes of GABAergic interneurons are reduced or deficient by non-cell-autonomous functions of GFRA1 in other factors of the olfactory system [62]. In addition, the upregulation of GDNF and GFRA1 has been reported in stroke (ischemia) and epilepsy (seizure disorder) [63,64]. In contrast, GDNF levels were found to be decreased in Alzheimer’s disease (AD), leading to progressive loss of cognitive function and dementia [65]. In fact, despite occurring in more than 10% of people after the age of 65, AD diagnosis in early stages is still difficult. Importantly, a recent study showed that GDNF was found as one of 18 signaling proteins identified in blood from 250 AD patients [66]. As mentioned earlier, recent AD studies suggest that GDNF increases the survival of cells by promoting the release of β-amyloid peptide from neuron-like cells, without increased expression of amyloid protein precursor (APP) or secretase [40,65]. Intriguingly, recent studies of neuropsychiatric disorders have reported that increased protein levels of GDNF or its polymorphism were observed in addiction, bipolar disorder, obsessive compulsive disorder, autism, schizophrenia, and attention deficit hyperactivity disorder [67,68,69,70,71]. In contrast, the protein levels of GDNF and its receptor GFRA1 were reduced in depression. In particular, downregulation of GFRA1a was associated with increased expression of microRNAs. Further studies showed that miR-511, which targets long 3′ untranslated region-containing transcripts coding for GFRA1a, repressed GFRA1a expression in human neural progenitor cells without altering GFRA1b expression, suggesting that microRNAs are involved in the modulation of GDNF/GFRA1 signaling [72]. 

Taken together, these findings demonstrate that clarification of the emerging roles of GDNF/GFRA1 signaling in CNS and PNS is essential to the development of biomarkers to accurately diagnose and drugs to treat these neuronal diseases.

## 4. GFRA1 in Cancer

Given the function of GFRA1 in neuronal development, GFRA1 has been studied with eyes on the emerging therapeutic treatment in neuronal disease, especially Parkinson’s disease. However, recent evidence suggests that GFRA1 is robustly involved in cancer progression [73]. Indeed, aberrant GDNF or GFRA1 expression has been often found in various cancer cells including those of the pancreas, skin and breast, as well is in osteosarcoma (Table 1). Recent studies provide evidence that GFRA1 is implicated in tumorigenesis and cancer progression. One research group showed that GFRA1 and RET are overexpressed in estrogen receptor-positive (ER+) tumors by screening a tissue microarray of invasive breast cancer. We further investigated whether tumor progression in these tumors is driven by the expression of GFRA1 and RET receptors or GDNF which is regulated in response to the inflammatory microenvironment surrounding many epithelial cancers. In tumor xenografts, they showed that the inflammatory cytokines tumor necrosis factor-α and interleukin-1β act synergistically to up-regulate GDNF expression and this effect is mediated by a GDNF/GFRA1/RET axis [74]. Another research group found that circular GFRA1 RNA (circGFRA1) regulates GFRA1 expression through sponging miR-34a to exert regulatory functions in triple negative breast cancer (TNBC) [75]. Their findings imply circGFRA1 may be a potential biomarker and/or therapeutic target for TNBC. Our study showed that GFRA1 expression is regulated by nuclear factor kappa B (NFκB) in pancreatic cancer. GDNF/GFRA1/RET signaling promotes cell proliferation and migration through SRC and MMP signaling [40]. Unlike neuronal cells, GFRA1 overexpression induces tumor progression in cancer cells. Collectively, the results from these investigations provide support to the idea that GFRA1 may be applicable as a biomarker and/or therapeutic target for cancer. Further understanding of GFRA1 expression will provide a major clue to solve the riddle of cell outcome, neuronal cell death and cancer cell proliferation and survival.

## 5. Emerging Roles of GFRA1 in Chemoresistance

Although osteosarcoma is not a common cancer, it is a serious disease given that the most affected age group are children and adolescents [80]. In addition, despite the fact that the long-term survival rate has increased to approximately 70% thanks to the use of adjuvant therapy, about 10–20% of patients have tumors which have already metastasized [81]. Indeed, it could be said that the success and draw-back of chemotherapy is dependent on the resistance of cells to the drug used. To date, cisplatin is the most widely used platinum-based anticancer drug for solid tumors [82]. It interacts with nucleophilic N7 sites of purine bases in DNA to induce DNA damage which leads to inhibition of tumor cell division and initiation of apoptosis, or programmed cell death [83]. However, due to the resistance of cells to cisplatin and drug toxicity, use of a combination therapy of methotrexate, doxorubicin and cisplatin has become common practice. Chemoresistance can be caused by the failure of drug export, DNA repair mechanisms, cancer stem cells, apoptotic signaling, or self-sufficiency for growth factor signaling [84]. Thus, understanding the mechanism of chemoresistance development may facilitate early detection, diagnosis and prognosis, thereby making it essential to treat cancer more effectively. 

**Figure 4:**
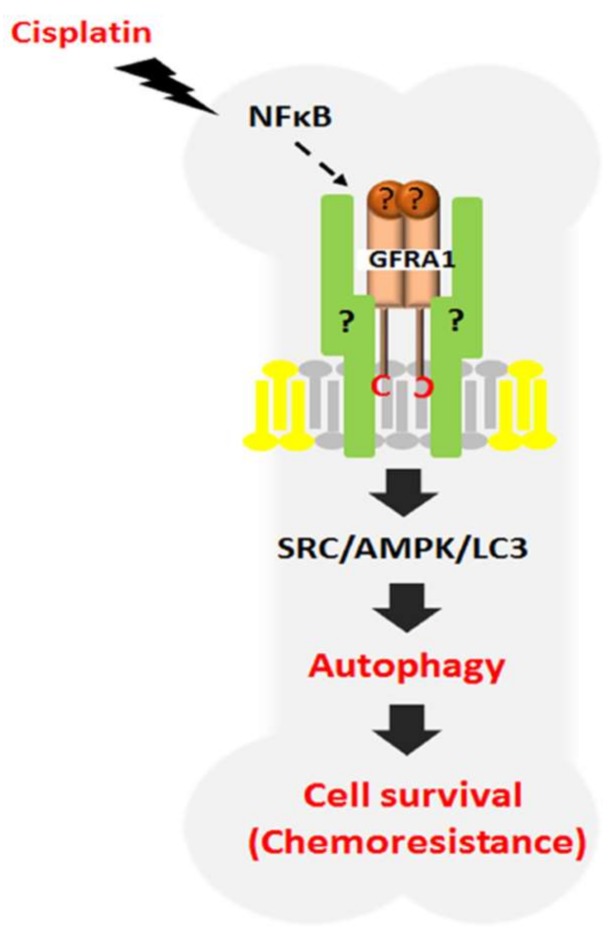
Schematic diagram for autophagy mediated by GFRA1 in cisplatin-resistant osteosarcoma. Cisplatin induces GFRA1 through NFκB, and GFRA1 activates autophagy by SRC/AMPK signaling. Finally, GFRA1-mediated autophagy inhibits cisplatin-induced apoptosis, resulting in cell proliferation and migration.

Autophagy is known as a self-digest mechanism for recycling the unnecessary and dysfunctional components to maintain homeostasis of cells [85,86]. At first, it was regarded as an alternative cell death mechanism known as programmed cell death II [87,88,89]. However, recent paradoxical findings demonstrated that autophagy also can play a role in cell survival against environmental stresses including nutrient deficiency, chemotherapy, radiation, and hypoxia [87,88,90]. Currently, the roles of autophagy in either apoptotic cell death as a tumor suppressive mechanism or cell survival mechanisms remain mostly controversial. Moreover, the regulatory mechanism of autophagy in chemoresistance has rarely been studied. Recently, our study demonstrated that GFRA1 induces autophagy as a novel regulatory mechanism of osteosarcoma chemoresistance; although neither ligand nor GFRA1 coreceptor were identified in this study, we found cisplatin triggers GFRA1 expression through NFκB [73]. The level of GFRA1 expression was increased in two osteosarcoma cell lines, MG-63 and U-2 OS, following treatment with cisplatin at both transcriptional and translational levels. However, two other chemotherapeutic drugs that were used for osteosarcoma treatment, doxorubicin and methotrexate, did not induce GFRA1 expression, indicating that cisplatin specifically induces GFRA1 expression. However, we do not exclude the possibility that other chemotherapeutic drugs can also induce GFRA1 expression in other types of cells as well as osteosarcoma cells. Previous studies have shown that NFκB upregulates GFRA1 mRNA expression through transcriptional activation [40]. Consistent with this finding, the levels of NFκB and phosphorylated NFκB expression were increased in osteosarcoma cells after cisplatin treatment compared to untreated cells. Inhibition of NFκB expression significantly reduced GFRA1 expression, suggesting that cisplatin-mediated activation of NFκB is one critical signaling pathway for GFRA1 expression [73]. Knockdown of GFRA1 expression in osteosarcoma cells significantly increased cisplatin-induced apoptosis, while overexpression of GFRA1 reduced cisplatin-induced apoptosis. Further studies showed that cisplatin treatment significantly increased autophagy in osteosarcoma cells, whereas it was not observed in GFRA1-deficient cells. GFRA1 overexpression in osteosarcoma cells significantly increased cell proliferation in the presence or absence of cisplatin with increased autophagy compared to control cells and this effect was blocked by the inhibition of autophagy. Src is one of the downstream targets of GFRA1 signaling [9]. Mechanistic studies showed that Src phosphorylation was increased in osteosarcoma cells with increased GFRA1 expression following cisplatin treatment and subsequently increased AMP-activated protein kinase (AMPK) phosphorylation, which is involved in the regulation of autophagy. Activated Src/AMPK signaling by GFRA1 increased the expression levels of beclin 1, high mobility group box 1 (HMGB1), and microtubule-associated protein light chain 3-II (LC3-II), which are downstream molecules involved in autophagy, suggesting that cisplatin-induced GFRA1 vertically transferred its survival signal to autophagy molecules through the Src/AMPK pathway (Figure 4). GDNF is the major ligand of GFRA1 as described above. However, GDNF had no effect on osteosarcoma cell survival after cisplatin treatment. This result implies that cisplatin-mediated activation of GFRA1 signaling is independent of GDNF. In addition, RET, a coreceptor of GDNF, was not expressed in osteosarcoma cells [73]. Mouse xenograft studies showed that mice injected with GFRA1-overexpressing osteosarcoma cells started to develop tumors 5 days after injection and produced large-sized tumors (~90 mm^3^). In contrast, mice injected with control osteosarcoma cells did not develop tumors until 17 days after injection and the resultant tumors were small (~10 mm^3^). Treatment of mice with chloroquine, an autophagic inhibitor, in combination with cisplatin significantly reduced tumor volume compared with PBS, chloroquine, or cisplatin-treated mice. Interestingly, we found that four out of nine osteosarcoma patients whose tumors were metastasized to the lung were positive for both GFRA1 and HMGB1 expression. These results showed that GFRA1-mediated autophagy is associated with cancer cell survival/tumor progression in patients that did not respond to cisplatin treatment [73]. In summary, our data showed that GFRA1 plays a critical role in cisplatin-induced chemoresistance via autophagy and it suggests that GFRA1 may be a potential therapeutic target against chemoresistance.

## 6. Conclusions

Although studies of GDNF family ligands and their receptors reveal that they are emerging targets for neuronal disease and cancer therapy, the complexity of their signaling still remains unclear. GFLs and GFRA1 expression levels may be dependent on their specific roles in cells. In addition, the choice between GDNF ligand family and GFRA1 also may determine their function. In other words, because cell-to-cell interaction is required for the communication of neuronal cells, GDNF/GFRA1 levels may be increased. Definitely, the discovery of sGFRA provides a clue as to how GDNF ligands and co-receptors can physiologically interact at a distance. The loss of GDNF/GFRA1 in the neuron inhibits its development, such as synapse formation in neuronal cells. Similarly, aberrant expression of GFRA1 has been often observed in cancer. Furthermore, GFRA1 expression is elevated by cisplatin treatment in osteosarcoma cells. GFRA1-mediated signaling activated autophagy, resulting in hyperproliferation, cell survival and metastasis in osteosarcoma. Taken together, a further understanding of the mechanisms involving GFRA1 may provide critical information towards discovering novel potential therapeutic approaches for overcoming chemoresistance in osteosarcoma.

## Figures and Tables

**Table 1:** The expression of GDNF ligands and its receptors in cancer cells.

Type of Ligand	Type of Receptor	Biological Functions	Type of Cancer	References
ARTN	GFRA1/GFRA3	ND	mammary carcinoma	[76]
GDNF	RET/GFRA1	ND	Breast cancer	[74]
GDNF	RET/GFRA1	Oncogenicity	malignant melanoma	[77]
GDNF	RET/GFRA1	Aromatase inhibitor resistance	Breast cancer	[78]
ND	GFRA1	Cisplatin resistance by autophagy	Osteosarcoma	[73]
ND	GFRA1	ND	Breast cancer	[79]
ND	GFRA1	ND	Breast cancer	[75]
GDNF	GFRA1	Cell migration and proliferation	Pancreatic cancer	[40]

ARTN, artemin; GDNF, glial cell-line-derived neurotrophic factor; GFRA1, GDNF receptor alpha 1; GFRA3, GDNF receptor alpha 3; RET, rearranged during transformation; ND, not determined.

## References

[1] Jing S., Wen D., Yu Y., Holst P.L., Luo Y., Fang M., Tamir R., Antonio L., Hu Z., Cupples R. (1996). GDNF-induced activation of the ret protein tyrosine kinase is mediated by GDNFR-α, a novel receptor for GDNF. Cell.

[2] Treanor J.J., Goodman L., de Sauvage F., Stone D.M., Poulsen K.T., Beck C.D., Gray C., Armanini M.P., Pollock R.A., Hefti F. (1996). Characterization of a multicomponent receptor for GDNF. Nature.

[3] Baloh R.H., Tansey M.G., Golden J.P., Creedon D.J., Heuckeroth R.O., Keck C.L., Zimonjic D.B., Popescu N.C., Johnson E.M., Milbrandt J. (1997). TRNR2, a novel receptor that mediates neurturin and GDNF signaling through Ret. Neuron.

[4] Baloh R.H., Gorodinsky A., Golden J.P., Tansey M.G., Keck C.L., Popescu N.C., Johnson E.M., Milbrandt J. (1998). GFRα3 is an orphan member of the GDNF/neurturin/persephin receptor family. Proc. Natl. Acad. Sci. USA.

[5] Baloh R.H., Tansey M.G., Lampe P.A., Fahrner T.J., Enomoto H., Simburger K.S., Leitner M.L., Araki T., Johnson E.M., Milbrandt J. (1998). Artemin, a novel member of the GDNF ligand family, supports peripheral and central neurons and signals through the GFRα3-RET receptor complex. Neuron.

[6] Naveilhan P., Baudet C., Mikaels A., Shen L., Westphal H., Ernfors P. (1998). Expression and regulation of GFRα3, a glial cell line-derived neurotrophic factor family receptor. Proc. Natl. Acad. Sci. USA.

[7] Enokido Y., de Sauvage F., Hongo J.A., Ninkina N., Rosenthal A., Buchman V.L., Davies A.M. (1998). GFRα-4 and the tyrosine kinase Ret form a functional receptor complex for persephin. Curr. Biol..

[8] Lin L.F., Doherty D.H., Lile J.D., Bektesh S., Collins F. (1993). GDNF: A glial cell line-derived neurotrophic factor for midbrain dopaminergic neurons. Science.

[9] Airaksinen M.S., Saarma M. (2002). The GDNF family: Signalling, biological functions and therapeutic value. Nat. Rev. Neurosci..

[10] Ibanez C.F., Andressoo J.O. (2017). Biology of GDNF and its receptors—Relevance for disorders of the central nervous system. Neurobiol. Dis..

[11] Ibanez C.F. (2013). Structure and physiology of the RET receptor tyrosine kinase. Cold Spring Harb Perspect. Biol..

[12] Trupp M., Arenas E., Fainzilber M., Nilsson A.S., Sieber B.A., Grigoriou M., Kilkenny C., Salazar-Grueso E., Pachnis V., Arumae U. (1996). Functional receptor for GDNF encoded by the c-ret proto-oncogene. Nature.

[13] Saarma M. (2000). GDNF—A stranger in the TGF-β superfamily?. Eur. J. Biochem..

[14] Sariola H., Saarma M. (2003). Novel functions and signalling pathways for GDNF. J. Cell Sci..

[15] Santoro M., Melillo R.M., Carlomagno F., Vecchio G., Fusco A. (2004). Minireview: RET: Normal and abnormal functions. Endocrinology.

[16] Paratcha G., Ledda F., Ibanez C.F. (2003). The neural cell adhesion molecule NCAM is an alternative signaling receptor for GDNF family ligands. Cell.

[17] Meng X., Lindahl M., Hyvonen M.E., Parvinen M., de Rooij D.G., Hess M.W., Raatikainen-Ahokas A., Sainio K., Rauvala H., Lakso M. (2000). Regulation of cell fate decision of undifferentiated spermatogonia by GDNF. Science.

[18] Viglietto G., Dolci S., Bruni P., Baldassarre G., Chiariotti L., Melillo R.M., Salvatore G., Chiappetta G., Sferratore F., Fusco A. (2000). Glial cell line-derived neutrotrophic factor and neurturin can act as paracrine growth factors stimulating DNA synthesis of Ret-expressing spermatogonia. Int. J. Oncol..

[19] Costantini F., Shakya R. (2006). GDNF/Ret signaling and the development of the kidney. Bioessays.

[20] Besset V., Scott R.P., Ibanez C.F. (2000). Signaling complexes and protein-protein interactions involved in the activation of the Ras and phosphatidylinositol 3-kinase pathways by the c-Ret receptor tyrosine kinase. J. Biol. Chem..

[21] Paratcha G., Ledda F., Baars L., Coulpier M., Besset V., Anders J., Scott R., Ibanez C.F. (2001). Released GFRalpha1 potentiates downstream signaling, neuronal survival, and differentiation via a novel mechanism of recruitment of c-Ret to lipid rafts. Neuron.

[22] Ledda F., Paratcha G., Ibanez C.F. (2002). Target-derived GFRalpha1 as an attractive guidance signal for developing sensory and sympathetic axons via activation of Cdk5. Neuron.

[23] Grumet M., Rutishauser U., Edelman G.M. (1982). Neural cell adhesion molecule is on embryonic muscle cells and mediates adhesion to nerve cells in vitro. Nature.

[24] Covault J., Sanes J.R. (1985). Neural cell adhesion molecule (N-CAM) accumulates in denervated and paralyzed skeletal muscles. Proc. Natl. Acad. Sci. USA.

[25] Thiery J.P., Duband J.L., Rutishauser U., Edelman G.M. (1982). Cell adhesion molecules in early chicken embryogenesis. Proc. Natl. Acad. Sci. USA.

[26] Rutishauser U., Thiery J.P., Brackenbury R., Edelman G.M. (1978). Adhesion among neural cells of the chick embryo. III. Relationship of the surface molecule CAM to cell adhesion and the development of histotypic patterns. J. Cell Biol..

[27] Noble M., Albrechtsen M., Moller C., Lyles J., Bock E., Goridis C., Watanabe M., Rutishauser U. (1985). Glial cells express N-CAM/D2-CAM-like polypeptides in vitro. Nature.

[28] Pollerberg E.G., Sadoul R., Goridis C., Schachner M. (1985). Selective expression of the 180-kD component of the neural cell adhesion molecule N-CAM during development. J. Cell Biol..

[29] Van Acker H.H., Anguille S., Willemen Y., Van den Bergh J.M., Berneman Z.N., Lion E., Smits E.L., Van Tendeloo V.F. (2016). Interleukin-15 enhances the proliferation, stimulatory phenotype, and antitumor effector functions of human gamma delta T cells. J. Hematol. Oncol..

[30] Kelly-Rogers J., Madrigal-Estebas L., O’Connor T., Doherty D.G. (2006). Activation-induced expression of CD56 by T cells is associated with a reprogramming of cytolytic activity and cytokine secretion profile in vitro. Hum. Immunol..

[31] Roothans D., Smits E., Lion E., Tel J., Anguille S. (2013). CD56 marks human dendritic cell subsets with cytotoxic potential. Oncoimmunology.

[32] Reyes A.A., Small S.J., Akeson R. (1991). At least 27 alternatively spliced forms of the neural cell adhesion molecule mRNA are expressed during rat heart development. Mol. Cell. Biol..

[33] Wang C.Y., Yang F., He X.P., Je H.S., Zhou J.Z., Eckermann K., Kawamura D., Feng L., Shen L., Lu B. (2002). Regulation of neuromuscular synapse development by glial cell line-derived neurotrophic factor and neurturin. J. Biol. Chem..

[34] Nielsen J., Gotfryd K., Li S., Kulahin N., Soroka V., Rasmussen K.K., Bock E., Berezin V. (2009). Role of glial cell line-derived neurotrophic factor (GDNF)-neural cell adhesion molecule (NCAM) interactions in induction of neurite outgrowth and identification of a binding site for NCAM in the heel region of GDNF. J. Neurosci..

[35] Ledda F. (2007). Ligand-induced cell adhesion as a new mechanism to promote synapse formation. Cell Adhes. Migr..

[36] Glerup S., Lume M., Olsen D., Nyengaard J.R., Vaegter C.B., Gustafsen C., Christensen E.I., Kjolby M., Hay-Schmidt A., Bender D. (2013). SorLA controls neurotrophic activity by sorting of GDNF and its receptors GFRα1 and RET. Cell Rep..

[37] Arenas E., Trupp M., Akerud P., Ibanez C.F. (1995). GDNF prevents degeneration and promotes the phenotype of brain noradrenergic neurons in vivo. Neuron.

[38] Gill S.S., Patel N.K., Hotton G.R., O’Sullivan K., McCarter R., Bunnage M., Brooks D.J., Svendsen C.N., Heywood P. (2003). Direct brain infusion of glial cell line-derived neurotrophic factor in Parkinson disease. Nat. Med..

[39] Lang A.E., Gill S., Patel N.K., Lozano A., Nutt J.G., Penn R., Brooks D.J., Hotton G., Moro E., Heywood P. (2006). Randomized controlled trial of intraputamenal glial cell line-derived neurotrophic factor infusion in Parkinson disease. Ann. Neurol..

[40] Kim M.H., Kim H.B., Acharya S., Sohn H.M., Jun J.Y., Chang I.Y., You H.J. (2009). Ape1/Ref-1 induces glial cell-derived neurotropic factor (GDNF) responsiveness by upregulating GDNF receptor alpha1 expression. Mol. Cell. Biol..

[41] Kang M.Y., Kim K.Y., Yoon Y., Kang Y., Kim H.B., Youn C.K., Kim D.H., Kim M.H. (2009). Ape1/Ref-1 Stimulates GDNF/GFRα1-mediated Downstream Signaling and Neuroblastoma Proliferation. Korean J. Physiol. Pharmacol..

[42] Newgreen D., Young H.M. (2002). Enteric nervous system: Development and developmental disturbances—Part 2. Pediatr. Dev. Pathol..

[43] Shen L., Pichel J.G., Mayeli T., Sariola H., Lu B., Westphal H. (2002). Gdnf haploinsufficiency causes Hirschsprung-like intestinal obstruction and early-onset lethality in mice. Am. J. Hum. Genet..

[44] Borghini S., Bocciardi R., Bonardi G., Matera I., Santamaria G., Ravazzolo R., Ceccherini I. (2002). Hirschsprung associated GDNF mutations do not prevent RET activation. Eur. J. Hum. Genet..

[45] Borrego S., Fernandez R.M., Dziema H., Niess A., Lopez-Alonso M., Antinolo G., Eng C. (2003). Investigation of germline GFRA4 mutations and evaluation of the involvement of GFRA1, GFRA2, GFRA3, and GFRA4 sequence variants in Hirschsprung disease. J. Med. Genet..

[46] Eketjall S., Ibanez C.F. (2002). Functional characterization of mutations in the GDNF gene of patients with Hirschsprung disease. Hum. Mol. Genet..

[47] Bespalov M.M., Sidorova Y.A., Tumova S., Ahonen-Bishopp A., Magalhaes A.C., Kulesskiy E., Paveliev M., Rivera C., Rauvala H., Saarma M. (2011). Heparan sulfate proteoglycan syndecan-3 is a novel receptor for GDNF, neurturin, and artemin. J. Cell Biol..

[48] Pozas E., Ibanez C.F. (2005). GDNF and GFRalpha1 promote differentiation and tangential migration of cortical GABAergic neurons. Neuron.

[49] Perrinjaquet M., Sjostrand D., Moliner A., Zechel S., Lamballe F., Maina F., Ibanez C.F. (2011). MET signaling in GABAergic neuronal precursors of the medial ganglionic eminence restricts GDNF activity in cells that express GFRα1 and a new transmembrane receptor partner. J. Cell Sci..

[50] Charoy C., Nawabi H., Reynaud F., Derrington E., Bozon M., Wright K., Falk J., Helmbacher F., Kindbeiter K., Castellani V. (2012). gdnf activates midline repulsion by Semaphorin3B via NCAM during commissural axon guidance. Neuron.

[51] Duveau V., Fritschy J.M. (2010). PSA-NCAM-dependent GDNF signaling limits neurodegeneration and epileptogenesis in temporal lobe epilepsy. Eur. J. Neurosci..

[52] Euteneuer S., Yang K.H., Chavez E., Leichtle A., Loers G., Olshansky A., Pak K., Schachner M., Ryan A.F. (2013). Glial cell line-derived neurotrophic factor (GDNF) induces neuritogenesis in the cochlear spiral ganglion via neural cell adhesion molecule (NCAM). Mol. Cell. Neurosci..

[53] Paratcha G., Ledda F. (2008). GDNF and GFRalpha: A versatile molecular complex for developing neurons. Trends Neurosci..

[54] Paratcha G., Ibanez C.F. (2002). Lipid rafts and the control of neurotrophic factor signaling in the nervous system: Variations on a theme. Curr. Opin. Neurobiol..

[55] Krakora D., Mulcrone P., Meyer M., Lewis C., Bernau K., Gowing G., Zimprich C., Aebischer P., Svendsen C.N., Suzuki M. (2013). Synergistic effects of GDNF and VEGF on lifespan and disease progression in a familial ALS rat model. Mol. Ther..

[56] Meissner W.G., Frasier M., Gasser T., Goetz C.G., Lozano A., Piccini P., Obeso J.A., Rascol O., Schapira A., Voon V. (2011). Priorities in Parkinson’s disease research. Nat. Rev. Drug Discov..

[57] Trupp M., Belluardo N., Funakoshi H., Ibanez C.F. (1997). Complementary and overlapping expression of glial cell line-derived neurotrophic factor (GDNF), c-ret proto-oncogene, and GDNF receptor-alpha indicates multiple mechanisms of trophic actions in the adult rat CNS. J. Neurosci..

[58] Burke R.E., O’Malley K. (2013). Axon degeneration in Parkinson’s disease. Exp. Neurol..

[59] Kholodilov N., Kim S.R., Yarygina O., Kareva T., Cho J.W., Baohan A., Burke R.E. (2011). Glial cell line-derived neurotrophic factor receptor-α1 expressed in striatum in trans regulates development and injury response of dopamine neurons of the substantia nigra. J. Neurochem..

[60] Pruett B.S., Salvatore M.F. (2013). Nigral GFRalpha1 infusion in aged rats increases locomotor activity, nigral tyrosine hydroxylase, and dopamine content in synchronicity. Mol. Neurobiol..

[61] Ledda F., Paratcha G., Sandoval-Guzman T., Ibanez C.F. (2007). GDNF and GFRα1 promote formation of neuronal synapses by ligand-induced cell adhesion. Nat. Neurosci..

[62] Marks C., Belluscio L., Ibanez C.F. (2012). Critical role of GFRalpha1 in the development and function of the main olfactory system. J. Neurosci..

[63] Duarte E.P., Curcio M., Canzoniero L.M., Duarte C.B. (2012). Neuroprotection by GDNF in the ischemic brain. Growth Factors.

[64] Reeben M., Laurikainen A., Hiltunen J.O., Castren E., Saarma M. (1998). The messenger RNAs for both glial cell line-derived neurotrophic factor receptors, c-ret and GDNFRalpha, are induced in the rat brain in response to kainate-induced excitation. Neuroscience.

[65] Scholz D., Chernyshova Y., Leist M. (2013). Control of Aβ release from human neurons by differentiation status and RET signaling. Neurobiol. Aging.

[66] Ray S., Britschgi M., Herbert C., Takeda-Uchimura Y., Boxer A., Blennow K., Friedman L.F., Galasko D.R., Jutel M., Karydas A. (2007). Classification and prediction of clinical Alzheimer’s diagnosis based on plasma signaling proteins. Nat. Med..

[67] Messer C.J., Eisch A.J., Carlezon W.A., Whisler K., Shen L., Wolf D.H., Westphal H., Collins F., Russell D.S., Nestler E.J. (2000). Role for GDNF in biochemical and behavioral adaptations to drugs of abuse. Neuron.

[68] Ubhi K., Inglis C., Mante M., Patrick C., Adame A., Spencer B., Rockenstein E., May V., Winkler J., Masliah E. (2012). Fluoxetine ameliorates behavioral and neuropathological deficits in a transgenic model mouse of α-synucleinopathy. Exp. Neurol..

[69] Tunca Z., Kivircik Akdede B., Ozerdem A., Alkin T., Polat S., Ceylan D., Bayin M., Cengizcetin Kocuk N., Simsek S., Resmi H. (2015). Diverse glial cell line-derived neurotrophic factor (GDNF) support between mania and schizophrenia: A comparative study in four major psychiatric disorders. Eur. Psychiatry.

[70] Fontenelle L.F., Barbosa I.G., Luna J.V., Rocha N.P., Silva Miranda A., Teixeira A.L. (2012). Neurotrophic factors in obsessive-compulsive disorder. Psychiatry Res..

[71] Moises H.W., Zoega T., Gottesman I.I. (2002). The glial growth factors deficiency and synaptic destabilization hypothesis of schizophrenia. BMC Psychiatry.

[72] Maheu M., Lopez J.P., Crapper L., Davoli M.A., Turecki G., Mechawar N. (2015). MicroRNA regulation of central glial cell line-derived neurotrophic factor (GDNF) signalling in depression. Transl. Psychiatry.

[73] Kim M., Jung J.Y., Choi S., Lee H., Morales L.D., Koh J.T., Kim S.H., Choi Y.D., Choi C., Slaga T.J. (2017). GFRA1 promotes cisplatin-induced chemoresistance in osteosarcoma by inducing autophagy. Autophagy.

[74] Esseghir S., Todd S.K., Hunt T., Poulsom R., Plaza-Menacho I., Reis-Filho J.S., Isacke C.M. (2007). A role for glial cell derived neurotrophic factor induced expression by inflammatory cytokines and RET/GFRα1 receptor up-regulation in breast cancer. Cancer Res..

[75] He R., Liu P., Xie X., Zhou Y., Liao Q., Xiong W., Li X., Li G., Zeng Z., Tang H. (2017). circGFRA1 and GFRA1 act as ceRNAs in triple negative breast cancer by regulating miR-34a. J. Exp. Clin. Cancer Res..

[76] Wu Z.S., Pandey V., Wu W.Y., Ye S., Zhu T., Lobie P.E. (2013). Prognostic significance of the expression of GFRα1, GFRalpha3 and syndecan-3, proteins binding ARTEMIN, in mammary carcinoma. BMC Cancer.

[77] Ohshima Y., Yajima I., Takeda K., Iida M., Kumasaka M., Matsumoto Y., Kato M. (2010). c-RET molecule in malignant melanoma from oncogenic RET-carrying transgenic mice and human cell lines. PLoS ONE.

[78] Morandi A., Martin L.A., Gao Q., Pancholi S., Mackay A., Robertson D., Zvelebil M., Dowsett M., Plaza-Menacho I., Isacke C.M. (2013). GDNF-RET signaling in ER-positive breast cancers is a key determinant of response and resistance to aromatase inhibitors. Cancer Res..

[79] Bhakta S., Crocker L.M., Chen Y., Hazen M., Schutten M.M., Li D., Kuijl C., Ohri R., Zhong F., Poon K.A. (2018). An anti-GDNF Family Receptor α 1(GFRA1) Antibody-Drug Conjugate for the Treatment of Hormone Receptor-Positive Breast Cancer. Mol. Cancer Ther..

[80] Ottaviani G., Jaffe N. (2009). The epidemiology of osteosarcoma. Cancer Treat. Res..

[81] Luetke A., Meyers P.A., Lewis I., Juergens H. (2014). Osteosarcoma treatment—Where do we stand? A state of the art review. Cancer Treat. Rev..

[82] Meyers P.A., Schwartz C.L., Krailo M., Kleinerman E.S., Betcher D., Bernstein M.L., Conrad E., Ferguson W., Gebhardt M., Goorin A.M. (2005). Osteosarcoma: A randomized, prospective trial of the addition of ifosfamide and/or muramyl tripeptide to cisplatin, doxorubicin, and high-dose methotrexate. J. Clin. Oncol..

[83] Dasari S., Tchounwou P.B. (2014). Cisplatin in cancer therapy: Molecular mechanisms of action. Eur. J. Pharmacol..

[84] Sui X., Chen R., Wang Z., Huang Z., Kong N., Zhang M., Han W., Lou F., Yang J., Zhang Q. (2013). Autophagy and chemotherapy resistance: A promising therapeutic target for cancer treatment. Cell Death Dis..

[85] Lum J.J., DeBerardinis R.J., Thompson C.B. (2005). Autophagy in metazoans: Cell survival in the land of plenty. Nat. Rev. Mol. Cell. Biol..

[86] Boya P., Reggiori F., Codogno P. (2013). Emerging regulation and functions of autophagy. Nat. Cell Biol..

[87] Kondo Y., Kanzawa T., Sawaya R., Kondo S. (2005). The role of autophagy in cancer development and response to therapy. Nat. Rev. Cancer.

[88] Levine B. (2006). Unraveling the role of autophagy in cancer. Autophagy.

[89] Mathew R., Karantza-Wadsworth V., White E. (2007). Role of autophagy in cancer. Nat. Rev. Cancer.

[90] Chen N., Karantza-Wadsworth V. (2009). Role and regulation of autophagy in cancer. Biochim. Biophys. Acta.

